# Effect of Greening Vacant Land on Mental Health of Community-Dwelling Adults: A Cluster Randomized Trial

**DOI:** 10.1001/jamanetworkopen.2018.0298

**Published:** 2018-07-20

**Authors:** Eugenia C. South, Bernadette C. Hohl, Michelle C. Kondo, John M. MacDonald, Charles C. Branas

**Affiliations:** 1 Department of Emergency Medicine, Perelman School of Medicine, University of Pennsylvania, Philadelphia; 2 Center for Emergency Care Policy and Research, Perelman School of Medicine, University of Pennsylvania, Philadelphia; 3 Department of Epidemiology, School of Public Health, School of Criminal Justice, Rutgers University, Newark, New Jersey; 4 Northern Research Station, Forest Service, US Department of Agriculture, Philadelphia, Pennsylvania; 5 Department of Criminology, School of Arts and Sciences, University of Pennsylvania, Philadelphia; 6 Department of Epidemiology, Mailman School of Public Health, Columbia University, New York, New York; 7 Department of Biostatistics and Epidemiology, Perelman School of Medicine, University of Pennsylvania, Philadelphia

## Abstract

**Importance:**

Neighborhood physical conditions have been associated with mental illness and may partially explain persistent socioeconomic disparities in the prevalence of poor mental health.

**Objective:**

To evaluate whether interventions to green vacant urban land can improve self-reported mental health.

**Design, Setting, and Participants:**

This citywide cluster randomized trial examined 442 community-dwelling sampled adults living in Philadelphia, Pennsylvania, within 110 vacant lot clusters randomly assigned to 3 study groups. Participants were followed up for 18 months preintervention and postintervention. This trial was conducted from October 1, 2011, to November 30, 2014. Data were analyzed from July 1, 2015, to April 16, 2017.

**Interventions:**

The greening intervention involved removing trash, grading the land, planting new grass and a small number of trees, installing a low wooden perimeter fence, and performing regular monthly maintenance. The trash cleanup intervention involved removal of trash, limited grass mowing where possible, and regular monthly maintenance. The control group received no intervention.

**Main Outcomes and Measures:**

Self-reported mental health measured by the Kessler-6 Psychological Distress Scale and the components of this scale.

**Results:**

A total of 110 clusters containing 541 vacant lots were enrolled in the trial and randomly allocated to the following 1 of 3 study groups: the greening intervention (37 clusters [33.6%]), the trash cleanup intervention (36 clusters [32.7%]), or no intervention (37 clusters [33.6%]). Of the 442 participants, the mean (SD) age was 44.6 (15.1) years, 264 (59.7%) were female, and 194 (43.9%) had a family income less than $25 000. A total of 342 participants (77.4%) had follow-up data and were included in the analysis. Of these, 117 (34.2%) received the greening intervention, 107 (31.3%) the trash cleanup intervention, and 118 (34.5%) no intervention. Intention-to-treat analysis of the greening intervention compared with no intervention demonstrated a significant decrease in participants who were feeling depressed (−41.5%; 95% CI, −63.6% to −5.9%; *P* = .03) and worthless (−50.9%; 95% CI, −74.7% to −4.7%; *P* = .04), as well as a nonsignificant reduction in overall self-reported poor mental health (−62.8%; 95% CI, −86.2% to 0.4%; *P* = .051). For participants living in neighborhoods below the poverty line, the greening intervention demonstrated a significant decrease in feeling depressed (−68.7%; 95% CI, −86.5% to −27.5%; *P* = .007). Intention-to-treat analysis of those living near the trash cleanup intervention compared with no intervention showed no significant changes in self-reported poor mental health.

**Conclusions and Relevance:**

Among community-dwelling adults, self-reported feelings of depression and worthlessness were significantly decreased, and self-reported poor mental health was nonsignificantly reduced for those living near greened vacant land. The treatment of blighted physical environments, particularly in resource-limited urban settings, can be an important treatment for mental health problems alongside other patient-level treatments.

**Trial Registration:**

isrctn.org Identifier: ISRCTN92582209

## Key Points

**Question:**

Does the greening of vacant urban land reduce self-reported poor mental health in community-dwelling adults?

**Findings:**

In this cluster randomized trial of urban greening and mental health, 110 randomly sampled vacant lot clusters were randomly assigned to 3 study groups. Among 342 participants included in the analysis, feeling depressed significantly decreased by 41.5% and self-reported poor mental health showed a reduction of 62.8% for those living near greened vacant lots compared with control participants.

**Meaning:**

The remediation of vacant and dilapidated physical environments, particularly in resource-limited urban settings, can be an important tool for communities to address mental health problems, alongside other patient-level treatments.

## Teaser

This cluster randomized trial compares the effects of greening vacant urban land (removing trash, grading land, planting grass and trees, installing fences, and regular maintenance) vs no intervention on the self-reported mental health of adults living in neighborhoods with vacant urban lots in Philadelphia, Pennsylvania.

## Introduction

Almost 1 in 5 US adults report some form of mental illness. Depression is the second largest contributor to years lived with disability in the United States,^1^ with more than 16 million adults experiencing an episode annually.^2,3^ Yet patient mental health services only account for an estimated 5% of total medical care spending in the United States.^4^ A broadening of treatment options to improve mental health is necessary, including interventions that fundamentally change harmful environmental surroundings that may be key contributors to mental illness.

Neighborhood physical conditions, including vacant or dilapidated spaces, trash, and lack of quality infrastructure such as sidewalks and parks, are associated with depression^5,6,7,8,9^ and are factors that may explain the persistent prevalence of mental illness in resource-limited communities.^10^ Vacant and dilapidated spaces are unavoidable neighborhood conditions that residents in low-resource communities encounter every day, making the very existence of these spaces a constant source of stress^11,12^ and possibly mental illness.

However, neighborhood physical conditions can also positively influence mental health.^13,14^ Spending time and living near green spaces have been associated with various improved mental health outcomes, including less depression, anxiety, and stress.^15,16,17,18,19^ Several studies have demonstrated a dose-response relationship between more time spent in green spaces and lower depression rates.^20,21^ Therefore, green space may be a potential buffer between inequitable neighborhood conditions and poor mental health outcomes.^22,23,24^

While patient-level therapies for mental illness will always be a vital aspect of treatment, changing the places where people live, work, and play may have broad population-level effects on mental health outcomes.^25^ There have been calls for the development of urban environmental interventions to improve mental health outcomes and well-being.^1,26^ In support of this, a number of observational studies have demonstrated the positive effect of vacant land greening interventions on urban health, crime, and stress.^12,27,28,29^ However, these prior studies have not been experimental and have not tested mental health outcomes. Given this, we evaluated data from, to our knowledge, the first citywide cluster randomized trial with the objective to test the effects of inexpensive, standardized, and reproducible vacant land remediation interventions—greening and trash cleanup—on health and safety. We report here on the mental health outcomes. Analysis of crime outcomes is reported elsewhere.^30^

## Methods

### Study Design

This citywide cluster randomized trial of a standardized, reproducible vacant lot greening intervention and vacant lot trash cleanup intervention was conducted in Philadelphia, Pennsylvania. The University of Pennsylvania institutional review board approved this trial. All participants provided written informed consent. All sections of this article were written using the Consolidated Standards of Reporting Trials (CONSORT) reporting guideline.^31^The trial protocol can be found in the Supplement.

### Vacant Lot Random Sampling and Random Assignment

A master list was compiled of all vacant lots citywide available from the city administrative records throughout January 2011. Vacant lots that were authorized by municipal ordinance as blighted and eligible for the intervention were randomly sampled for the trial. Eligible lots were included if they specifically (1) had existing violations signaling blight, including illegal dumping, abandoned cars, and/or unmanaged vegetation growth; and (2) had been abandoned, as confirmed through contact with the owner of record who, within a 10-day period, either authorized the intervention or did not reply. Owners included the city itself for publicly owned lots. We excluded lots that had insufficient blight or lack of abandonment, lots that were greater than 5500 sq ft, and lots that were fully paved parking lots.

Vacant lot clusters served as the intervention unit for the study. To form these clusters, the master list of eligible vacant lots was ordered based on the assignment of random numbers within 4 sections of the city.^32^ In each section of the city, the first vacant lot in the randomly ordered list was chosen as an index lot and a 0.25-mile radius buffer was created around that lot. All other eligible vacant lots on the master list that fell within this radius were used to form a cluster grouping of geographically proximal vacant lots that summed between 4500 to 5500 total sq ft; these lots were then removed from consideration as future index lots. This process then cycled to the next randomly ordered index lot on the list that was at least 0.25 miles away from the edge of prior clusters until all clusters were formed. This process guaranteed that no clusters overlapped, reducing potential spillover and contamination effects across trial arms.

**Figure 1.**
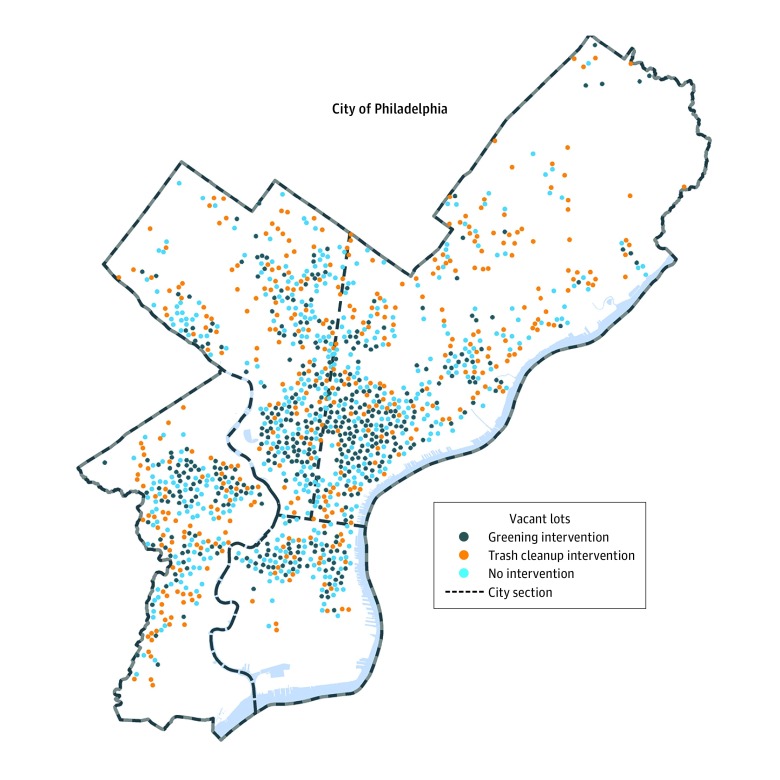
Distribution of Study Vacant Lots Across Philadelphia, Pennsylvania This map shows the distribution of randomly selected study vacant lots across 3 groups of the trial: the greening intervention, the trash cleanup intervention, and no intervention. The distribution of vacant lots shown is representative of those in the study, although for the purposes of confidentiality are not the locations of actual study lots.

Within each city section, clusters were randomly assigned to 1 of 3 study groups—the greening intervention, trash cleanup intervention, or no intervention (Figure 1). A repeated randomization procedure^33^ was used under a predetermined protocol that permitted repeated random allocation of the 3 study groups until a statistically significant balance was achieved with a set of potential confounding variables, including the total area and mean separating distance of the study vacant lots, the total vacant lots, resident population, and number of serious crimes (part I violent and property crimes), in each cluster.

### Vacant Lot Interventions and Control Group

**Figure 2.**
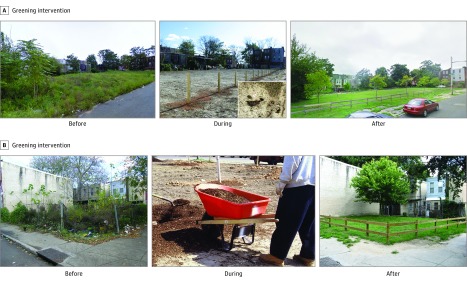
Vacant Lot Main Greening Intervention Images show blighted preperiod conditions and remediated postperiod restorations. A, The image shows the grass seeding method used to rapidly complete the treatment process. B, The after image shows the low wooden perimeter fence. Vacant lots shown here are representative of those in the study, although for purposes of confidentiality are not actual study lots.

The vacant lot greening intervention involved the cleaning and greening of vacant lots via a standard, reproducible process of removing trash and debris, grading the land, planting new grass and a small number of trees, installing a low wooden perimeter fence with openings, and performing regular maintenance (Figure 2). The vacant lot trash cleanup intervention group involved removal of trash and debris, limited grass mowing on the lot where possible, and regular maintenance. The Pennsylvania Horticultural Society designed and carried out the interventions over a 2-month period, from April 1, 2013, to May 31, 2013, followed by monthly maintenance. At the end of the postintervention period, vacant lots assigned to the control condition were scheduled for cleaning and greening.

### Random Sampling of Participants

Two preintervention interview survey waves were conducted from October 1, 2011, to March 31, 2013, and 2 postintervention survey waves were conducted from June 1, 2013, to November 30, 2014, with a sample of residents from each cluster. All participants completed at least 1 preintervention survey and 1 postintervention survey. The outer-bounding polygon and its centroid were calculated for each grouping of vacant lots per cluster. This centroid represented the point location that was mathematically closest to all the study vacant lots in each cluster. The address of the closest building to this point location was then determined as the starting point for house-to-house random sampling and enrollment of survey participants. At each starting address, a 2-person survey team walked in a predetermined random direction on the corresponding city block followed by randomly chosen adjacent city blocks within the cluster until a total of 5 participants had been identified, consented, and were interviewed. Both the survey team and participants were blinded to cluster intervention. Participants were told the study was about improving our understanding of urban health. One participant per household was chosen; in households with multiple eligible participants, the individual with the most recent birthday was chosen. All baseline interviews and most follow-up interviews were conducted in person; a handful of follow-up interviews were conducted by telephone. Both English-speaking and Spanish-speaking individuals 18 years and older were administered the survey in the language of their choice; only 2 Spanish-language surveys were administered. Each participant was compensated $25 per interview, which took an average of 39.6 minutes to complete. Based on the American Association for Public Opinion Research response rate calculator, our survey response rate was 47.4%.^34^ Our response rate matched or exceeded that of other surveys and was high enough to produce a reasonably representative sample of our target population.^35,36,37^

### Outcome Measures

At each interview, participants responded to questions about their perceptions of mental health, focusing on their experiences within the past 30 days to anchor responses in time relative to the intervention period and to avoid telescoping and overestimation. We used the validated short-form Kessler-6 Psychological Distress Scale (K6), a widely used community screening tool. The K6 was designed to evaluate the prevalence of serious mental illness in the community and does not make a clinical diagnosis of mental illness. Participants were asked to indicate how often they felt nervous, hopeless, restless, depressed, that everything was an effort, and worthless using the following scale: all of the time, most of the time, more than half of the time, less than half of the time, some of the time, or at no time. In keeping with the K6 order and scoring, the 2 middle categories were combined to create a score of 0 to 4 for each marker, which was then summed for a total score of 0 to 24. Using standard scoring guidelines, a score of 13 or greater indicated higher prevalence of serious mental illness or what we call *self-reported poor mental health*.^38,39^ Participants self-reported their race and/or ethnicity.

### Statistical Analysis

Prior to the study, sample size was determined by taking into account anticipated intracluster correlation, participant response prevalence, number of crimes reported to the police in each area, effect size, and power. The minimally detectable effect size, given 80% power and 4 time points based on the group before vs after interaction test for any pairwise comparison among the randomly allocated groups of lots, was calculated.^40^ From this, and predicting a 25% loss-to-follow-up rate, we estimated that we would maintain more than 80% power if we randomly surveyed 3 people per cluster twice before and twice after the intervention.

Intention-to-treat analyses of participants were conducted according to the randomly assigned vacant lot cluster intervention group in which they lived. Pairwise comparisons were completed for all study outcomes between the greening intervention group and the no intervention group as well as the trash cleanup intervention group and the no intervention group. These pairwise comparisons were tested for statistical significance (all tests were 2-sided and statistical significance was defined as *P* ≤ .05) using unadjusted random-effects, cross-sectional time series regressions that accounted for the cluster design of the trial. Random-effects regressions were chosen because we assumed that unobserved lot-specific effects were correlated over time at the cluster level. All statistical analyses were conducted using Stata, version 14.1 (StataCorp LLC).

Difference-in-differences analyses were calculated as interaction terms of 1-0 intervention-control differences multiplied by 0-1 pre-post differences. These difference-in-differences interaction terms were the primary independent variables of interest interpreted as the true effect of the interventions on the outcomes studied. The estimates from the difference-in-differences analysis were then divided by the overall magnitude of occurrence for each outcome in the intervention group to obtain percentage reductions.^27,29,41^ Additional subset analyses were also completed by neighborhood poverty levels using the census tracts within which study participants lived. The poverty threshold for 2013 was determined to be $19 530 per the average size of persons per household in Philadelphia and the 2013 poverty guidelines from the US Census Bureau and the Department of Health and Human Services Office of the Assistant Secretary for Planning and Evaluation.^42^

## Results

### Vacant Lots and Clusters

**Figure 3.**
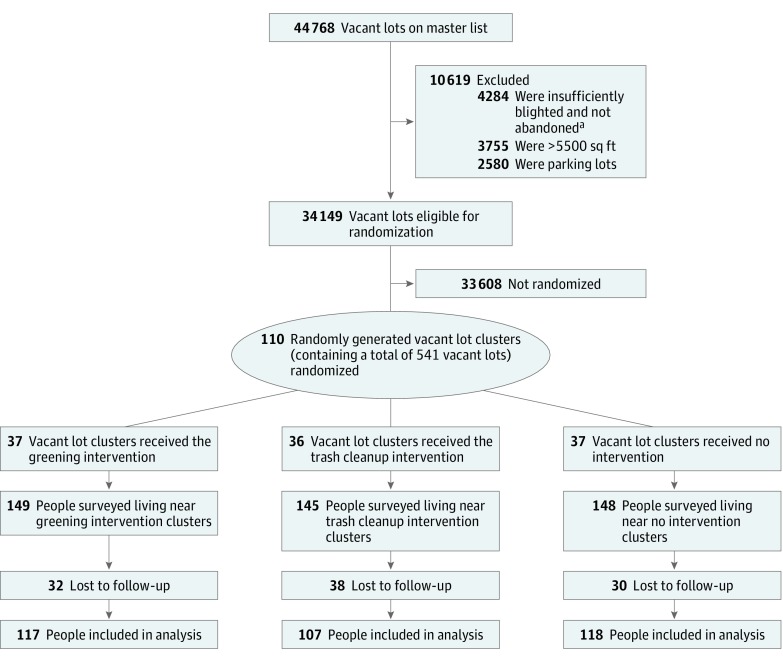
Flowchart of Vacant Lots and Participants Through Vacant Lot Greening Trial ^a^Vacant lots were classified as blighted if they (1) had existing violations signaling blight, including illegal dumping, abandoned cars, and/or unmanaged vegetation growth; and (2) had been abandoned, as confirmed through contact with the owner of record who, within a 10-day period, either authorized the intervention or did not reply. Those excluded as having insufficient blight or not confirmed as abandoned did not meet these conditions.

The master list included 44 768 vacant lots, 34 149 (76.3%) of which were deemed eligible for inclusion in the study. Ineligible lots were excluded owing to insufficient blight or not being abandoned (4284), being greater than 5500 sq ft (3755), and being existing private or commercial parking lots (2580). A total of 110 clusters containing 541 vacant lots were enrolled in the trial and randomly allocated to the following 1 of 3 study arms: the greening intervention (37 clusters [33.6%]), the trash cleanup intervention (36 clusters [32.7%]), or no intervention (37 clusters [33.6%]) (Figure 3). Of the clusters, 47 (42.7%) were included in neighborhood poverty subset analysis.

Balance was evident at the cluster level between the 3 intervention conditions in terms of total number of study lots per study arm (range, 161-206 lots), the mean number of study lots per cluster (range, 4.5-5.4 lots), the total square footage of study lots per cluster (range, 4844-4935 sq ft), the mean number of residents per cluster (range, 285-297 people), and the mean number of serious crimes, as reported by the Philadelphia Police Department, occurring within each cluster during the 18-month baseline period (range, 16.5-18.3 crimes) (Table 1).

**Table 1.** Baseline Characteristics Demonstrating Balance Across Study Groups^a^

Characteristic	No. (%)		
	Greening Intervention	Trash Cleanup Intervention	No Intervention Control
Vacant lot clusters			
No.	37	36	37
Resident population, mean (SD), No.	287.8 (117.5)	297.0 (124.6)	284.9 (130.5)
Serious crimes, mean (SD), No.^b^	16.5 (6.4)	18.3 (9.6)	17.1 (8.4)
Total eligible vacant lots, mean (SD), No.	38.3 (25.2)	43.1 (28.4)	38.1 (31.1)
Prior treated lots, mean (SD), No.	6.7 (9.5)	5.3 (9.7)	5.6 (14.1)
Total study lots, No.	206	174	161
Study lots per cluster, mean, No.	5.4	4.8	4.5
Study lots total area, mean (SD), sq ft	4844 (970.2)	4935 (991.6)	4872 (1375.7)
Study lots separation, mean (SD), ft	75.6 (85.5)	71.3 (77.3)	73.5 (70.2)
Participants			
No.	149	145	148
Age, mean (SD), y	43.3 (14.9)	44.2 (15.7)	45.3 (14.8)
Tenure in home, mean (SD), y	12.0 (14.1)	13.7 (15.8)	12.5 (14.4)
Sex			
Male	57 (38.3)	54 (37.2)	67 (45.3)
Female	92 (61.7)	91 (62.8)	81 (54.7)
Race/ethnicity			
White	12 (8.0)	14 (9.7)	21 (14.2)
Black	118 (79.2)	117 (80.7)	102 (68.9)
Other	20 (13.4)	15 (10.7)	23 (15.2)
Hispanic	14 (9.4)	12 (8.3)	17 (11.5)
Education			
Less than high school	34 (22.8)	44 (30.3)	31 (20.9)
High school	71 (47.7)	64 (44.1)	72 (48.7)
Any college	42 (28.2)	36 (24.8)	44 (29.7)
Employment status			
Employed	95 (63.8)	99 (68.3)	104 (70.3)
Unemployed	54 (36.2)	46 (31.7)	44 (29.7)
Family income, $			
<10 000	35 (23.5)	36 (24.8)	38 (25.7)
10 000 to <25 000	26 (17.5)	32 (22.1)	27 (18.2)
25 000 to <50 000	27 (18.1)	19 (13.1)	18 (12.2)
>50 000	8 (5.4)	8 (5.5)	16 (10.8)

^a^ Percentages may not total 100% because of nonresponse on specific variables.

^b^ Serious crimes include part I violent and property crimes.

### Participant Baseline Characteristics

Of the 442 participants, the mean (SD) age was 44.6 (15.1) years, 264 (59.7%) were female, and 194 (43.9%) had a family income less than $25 000. A total of 442 participants were interviewed during the preintervention period, and 342 (77.4%) of these original participants were interviewed during the postintervention period and are included in this analysis. This amounted to a 22.6% loss to follow-up; of the 100 lost participants, 78% could not be found in their original cluster, and 22% refused to participate in subsequent waves. Of the 442 participants, 149 (33.7%) were assigned to the greening intervention, 145 (32.8%) to the trash cleanup intervention, and 148 (33.5%) to no intervention. Of the 342 participants included in the analysis, 117 (34.2%) received the greening intervention, 107 (31.3%) the trash cleanup intervention, and 118 (34.5%) no intervention. A total of 139 people (40.6%) were included in the neighborhood poverty subset analyses, including 45 (32.4%) receiving the greening intervention, 51 (36.7%) the trash cleanup intervention, and 43 (30.9%) no intervention. Participant demographic characteristics were balanced between the 3 study arms, including mean tenure in the home (range, 12.0-13.7 years), mean age (range, 43.3-45.3 years), and percentage with family income less than $25 000 (range, 41.0%-46.9%) (Table 1).

### Participant-Reported Mental Health Outcomes

Intention-to-treat analyses demonstrated significant changes in participant-reported mental health outcomes. Intention-to-treat analyses of the greening intervention compared with no intervention demonstrated a significant decrease in feeling depressed (−41.5%; 95% CI, −63.6% to −5.9%; *P* = .03) and feeling worthless (−50.9%; 95% CI, −74.7% to −4.7%; *P* = .04). Analysis also demonstrated a nonsignificant reduction in overall self-reported poor mental health (−62.8%; 95% CI, −86.2% to 0.4%; *P* = .051), as calculated by the K6 (Table 2). When looking only at neighborhoods below the poverty line, feeling depressed significantly decreased (−68.7%; 95% CI, −86.5% to −27.5%; *P* = .007). There was no significant difference in self-reported poor mental health in neighborhoods below the poverty line.

**Table 2.** Intention-to-Treat Analyses of Vacant Lot Interventions and Self-reported Mental Health Outcomes

Response^a^	No Intervention		Greening Intervention				Trash Cleanup Intervention			
	Preperiod, %	Postperiod, %	Preperiod, %	Postperiod, %	Pre and Post Change vs Control, % (95% CI)	*P* Value	Preperiod, %	Postperiod, %	Pre and Post Change vs Control, % (95% CI)	*P* Value
All neighborhoods										
Nervous	27.9	23.8	34.0	23.0	−16.4 (−43.1 to 22.9)	.36	29.8	20.6	−11.7 (−41.6 to 33.6)	.56
Hopeless	13.2	8.7	16.4	8.9	−17.0 (−49.2 to 35.6)	.46	15.3	12.7	12.7 (−31.1 to 84.2)	.63
Restless	22.8	20.8	30.3	17.5	−33.1 (−55.8 to 1.2)	.06	22.6	19.7	−27.8 (−51.5 to 7.5)	.11
Depressed	11.8	8.7	15.2	10.5	−41.5 (−63.6 to −5.9)	.03	14.9	14.8	−15.4 (−49.5 to 41.9)	.53
Everything an effort	33.8	26.0	41.0	31.1	−7.6 (−41.3 to 45.4)	.73	39.5	31.6	−7.7 (−36.5 to 34.2)	.68
Worthless	6.6	8.7	10.3	5.1	−50.9 (−74.7 to −4.7)	.04	9.7	9.2	−27.6 (−65.0 to 49.6)	.38
Poor mental health^b^	5.5	4.8	9.4	3.9	−62.8 (−86.2 to 0.4)	.051	7.3	4.8	−30.1 (−74.7 to 93.2)	.49
Neighborhoods below poverty level^c^										
Nervous	32.1	26.6	39.5	19.4	−39.6 (−71.9 to 30.0)	.20	27.9	22.3	−34.8 (−39.7 to 57.0)	.30
Hopeless	17.9	10.9	18.5	6.0	−45.3 (−78.5 to 39.1)	.21	22.1	13.8	−33.7 (−69.5 to 44.0)	.30
Restless	28.6	23.4	33.3	23.4	−45.1 (−77.3 to 32.7)	.18	20.9	18.4	−15.6 (−54.9 to 58.0)	.60
Depressed	11.9	7.8	22.2	8.9	−68.7 (−86.5 to −27.5)	.007	19.8	19.5	−18.7 (−60.8 to 68.6)	.58
Everything an effort	40.5	31.2	42.0	26.9	−38.4 (−73.1 to 40.8)	.25	37.2	33.3	−8.1 (−46.5 to 58.0)	.76
Worthless	7.1	9.4	13.6	4.5	−52.6 (−86.6 to 67.5)	.25	14.0	10.4	−34.4 (−79.9 to 114.1)	.49
Poor mental health^b^	7.1	6.3	13.6	4.5	−76.7 (−96.2 to 44.8)	.12	11.6	6.9	−45.4 (−84.4 to 91.6)	.35

^a^ Participants focused on their experiences within the past 30 days. Possible responses were all of the time, most of the time, more than half of the time and/or less than half of the time, some of the time, or at no time; percentages are the proportion of participants responding “less than half the time” or “more often.”

^b^ Kessler-6 Psychological Distress Scale mental illness score ranged from 0 to 24, with each of the 6 components ranging from 0 to 4; scores of 13 or greater indicated poor self-reported mental health.

^c^ Neighborhood poverty levels were determined using the census tracts within which study participants lived.

Intention-to-treat analyses of the trash cleanup intervention compared with no intervention did not show any statistically significant differences between self-reported poor mental health measured by the K6 (Table 2). There was also no difference between groups for the individual components of the K6. The analysis of neighborhoods below the poverty line also did not indicate any difference in self-reported mental health between the groups.

## Discussion

In this citywide cluster randomized trial of 2 vacant land remediation interventions, greening was associated with a significant reduction in feeling depressed and worthless as well as a nonsignificant reduction in overall self-reported poor mental health for randomly sampled residents living nearby. The trash cleanup intervention was not associated with a reduction in feeling depressed or self-reported poor mental health.

To our knowledge, this is the first citywide cluster randomized trial of actual place-based changes to urban spaces. These results add much needed experimental evidence to a growing body of literature calling for structural changes to neighborhoods as a method for improving health and safety.^43,44^ This study extends previous work showing a clear association between green space and mental illness,^13,14,15,16,17,18,19,20,21^ by demonstrating that adding green space to people’s neighborhood environment can improve the trajectory of their mental health. Additionally, vacant lot greening is a relatively low-cost intervention (approximately $1597 per vacant lot and $180 per year to maintain) that we have previously shown to be a cost-beneficial solution to firearm violence.^29^ For these reasons, vacant lot greening may be an extremely attractive intervention for policy makers seeking to address urban blight.

Our findings indicate that the effect of vacant lot greening on feeling depressed was slightly stronger for those living in neighborhoods below the poverty line. Urban blight is an environmental condition that disproportionately affects low-resource neighborhoods, as evidenced by the fact that almost half of our participants had yearly family incomes less than $25 000. Making structural changes to the lowest-resource neighborhoods can make them healthier and may be an important mechanism to address persistent and entrenched socioeconomic health disparities.^45^

There are several possible mechanisms through which the vacant lot greening intervention but not the trash cleanup intervention improved feeling depressed and self-reported poor mental health. One significant difference between the 2 interventions was the creation of new green space. Green space, particularly in urban environments more likely to have a dearth of vegetation, has been linked to recovery from mental fatigue,^46^ a state of inattentiveness and irritability resulting from the information-processing demands of daily life. Spending time in or near nature can combat mental fatigue because it allows engagement without paying explicit attention.^46,47,48^ A related concept is the association between spending time in or near green space and stress reduction,^18,49^ which may in turn reduce mental illness. For example, walking past green space has been associated with reduction in heart rate,^12^ one marker of acute physiological stress.

Additionally, the presence of green space is associated with improved neighborhood social milieu, including the concepts of social cohesion, social capital, and collective efficacy.^50,51,52,53^ The presence of grass and trees is related to use of outdoor space and increased social activity that takes place in those outdoor spaces.^54^ Improved social conditions are, in turn, associated with better mental health.^55,56^ For example, living in a low-income neighborhood is associated with worse mental health indicators for people with low but not high social cohesion.^57^ Studies have found that social cohesion mediated a positive green space–mental health relationship.^58,59,60^ Additionally, previous studies have demonstrated an association of vacant lot greening with increased feelings of safety and decreased violent crime, both of which may work to improve mental illness.^27,28^ Fear of crime, for example, is associated with almost 2-fold higher likelihood of having depression.^61^

The other significant difference between the greening and trash cleanup interventions was the presence of a simple wooden post and rail fence. The fence delineates the newly greened space as one that is cared for but does have openings to indicate that people can enter the space. The fence is also meant to deter illegal dumping. Previous qualitative work conducted by our team indicated that vacant land causes people to feel stigmatized and abandoned by their community and government.^11^ Countering this with clear signs of neighborhood investment, such as a clearly marked newly greened vacant lot, may contribute to the improvements seen in feeling depressed and self-reported poor mental health.

### Limitations

There were several limitations to this study. We used the K6 to measure our outcome of interest and mental health. While this is a validated and widely used scale, it is still a single scale, and other mental illness screening and diagnosis tools and scales may produce different results. Furthermore, we did not conduct a *Diagnostic and Statistical Manual of Mental Disorders, Fifth Edition*–level diagnosis of mental illness but rather used a community screening tool. Another limitation is the duration of our study and loss to follow-up. We followed up people for 18 months following the blight remediation interventions and are unable to know if the effect of the interventions on mental health outcomes persisted past the study period. We also made every effort to minimize loss to follow-up of our study participants after they were first enrolled, although differential, nonrandom dropout in our 3 study arms and across all study waves could have affected our results. Finally, we did not specifically track if and how study participants used (or did not use) study vacant lots, although prior work has demonstrated signs of use, such as barbeques or chairs on similar vacant lots.^62^

## Conclusions

Among community-dwelling adults, self-reported feelings of depression and worthlessness were significantly decreased and self-reported poor mental health was nonsignificantly reduced for those living near greened vacant lots compared with control lots. The treatment of dilapidated physical environments can be an important tool for communities to address persistent mental health problems. These findings provide support to health care clinicians concerned with positively transforming the often chaotic and harmful environments that affect their patients. Our findings also offer evidence to policy makers interested in increasing municipal investments in the remediation of blighted urban spaces as an inexpensive^29^ and scalable way to improve mental health, particularly in low-resource neighborhoods.

## Notes

Supplement. **Trial Protocol**

## References

[1] Murray CJL, Atkinson C, Bhalla K, ; US Burden of Disease Collaborators The state of US health, 1990-2010: burden of diseases, injuries, and risk factors. JAMA. 2013;310(6):-. doi:10.1001/jama.2013.13805 23842577 PMC5436627

[2] US Center for Behavioral Health Statistics and Quality Key substance use and mental health indicators in the United States: results from the 2015 National Survey on Drug Use and Health. http://www.samhsa.gov/data/. Accessed November 13, 2017.

[3] Kessler RC, Berglund P, Demler O, Jin R, Merikangas KR, Walters EE Lifetime prevalence and age-of-onset distributions of *DSM-IV* disorders in the National Comorbidity Survey Replication. Arch Gen Psychiatry. 2005;62(6):593-602. doi:10.1001/archpsyc.62.6.593 15939837

[4] Kamal R, Cox C, Rousseau D; Kaiser Family Foundation Costs and outcomes of mental health and substance use disorders in the US. JAMA. 2017;318(5):415. doi:10.1001/jama.2017.8558 28763535

[5] Mair C, Diez Roux AV, Morenoff JD. Neighborhood stressors and social support as predictors of depressive symptoms in the Chicago Community Adult Health Study. Health Place. 2010;16(5):811-819. doi:10.1016/j.healthplace.2010.04.006 20434941 PMC2918682

[6] Latkin CA, Curry AD Stressful neighborhoods and depression: a prospective study of the impact of neighborhood disorder. J Health Soc Behav. 2003;44(1):34-44. doi:10.2307/1519814 12751309

[7] Hill TD, Ross CE, Angel RJ. Neighborhood disorder, psychophysiological distress, and health. J Health Soc Behav. 2005;46(2):170-186. doi:10.1177/002214650504600204 16028456

[8] Mair C, Diez Roux AV, Galea S Are neighbourhood characteristics associated with depressive symptoms? a review of evidence. J Epidemiol Community Health. 2008;62(11):940-946. 18775943 10.1136/jech.2007.066605

[9] Mair C, Diez Roux AV, Shen M, . Cross-sectional and longitudinal associations of neighborhood cohesion and stressors with depressive symptoms in the Multiethnic Study of Atherosclerosis. Ann Epidemiol. 2009;19(1):49-57. doi:10.1016/j.annepidem.2008.10.002 19064189 PMC2763272

[10] Hoebel J, Maske UE, Zeeb H, Lampert T Social inequalities and depressive symptoms in adults: the role of objective and subjective socioeconomic status. PLoS One. 2017;12(1):e0169764. doi:10.1371/journal.pone.0169764 28107456 PMC5249164

[11] Garvin E, Branas C, Keddem S, Sellman J, Cannuscio C More than just an eyesore: local insights and solutions on vacant land and urban health. J Urban Health. 2013;90(3):412-426. doi:10.1007/s11524-012-9782-7 23188553 PMC3665973

[12] South EC, Kondo MC, Cheney RA, Branas CC. Neighborhood blight, stress, and health: a walking trial of urban greening and ambulatory heart rate. Am J Public Health. 2015;105(5):909-913. doi:10.2105/AJPH.2014.302526 25790382 PMC4386540

[13] James P, Banay RF, Hart JE, Laden F A review of the health benefits of greenness. Curr Epidemiol Rep. 2015;2(2):131-142. doi:10.1007/s40471-015-0043-7 26185745 PMC4500194

[14] Seymour V The human-nature relationship and its impact on health: a critical review. Front Public Health. 2016;4:260. doi:10.3389/fpubh.2016.00260 27917378 PMC5114301

[15] Beyer KM, Kaltenbach A, Szabo A, Bogar S, Nieto FJ, Malecki KM Exposure to neighborhood green space and mental health: evidence from the Survey of the Health of Wisconsin. Int J Environ Res Public Health. 2014;11(3):3453-3472. doi:10.3390/ijerph110303453 24662966 PMC3987044

[16] McEachan RRC, Prady SL, Smith G, The association between green space and depressive symptoms in pregnant women: moderating roles of socioeconomic status and physical activity. J Epidemiol Community Health. 2016:70(3):253-259. doi:10.1136/jech-2015-205954 26560759 PMC4789818

[17] Wu Y-T, Prina AM, Jones A, Matthews FE, Brayne C; The Medical Research Council Cognitive Function and Ageing Studies Older people, the natural environment and common mental disorders: cross-sectional results from the Cognitive Function and Ageing Study. BMJ Open. 2015;5(9):e007936. doi:10.1136/bmjopen-2015-007936 26377504 PMC4577935

[18] Roe JJ, Thompson CW, Aspinall PA, . Green space and stress: evidence from cortisol measures in deprived urban communities. Int J Environ Res Public Health. 2013;10(9):4086-4103. doi:10.3390/ijerph10094086 24002726 PMC3799530

[19] Morita E, Fukuda S, Nagano J, . Psychological effects of forest environments on healthy adults: Shinrin-yoku (forest-air bathing, walking) as a possible method of stress reduction. Public Health. 2007;121(1):54-63. doi:10.1016/j.puhe.2006.05.024 17055544

[20] Cox DTC, Shanahan DF, Hudson HL, . Doses of nearby nature simultaneously associated with multiple health benefits. Int J Environ Res Public Health. 2017;14(2):172. doi:10.3390/ijerph14020172 28208789 PMC5334726

[21] Shanahan DF, Bush R, Gaston KJ, . Health benefits from nature experiences depend on dose. Sci Rep. 2016;6:28551. doi:10.1038/srep28551 27334040 PMC4917833

[22] Mitchell R, Popham F Effect of exposure to natural environment on health inequalities: an observational population study. Lancet. 2008;372(9650):1655-1660. doi:10.1016/S0140-6736(08)61689-X 18994663

[23] Hartig T Green space, psychological restoration, and health inequality. Lancet. 2008;372(9650):1614-1615. doi:10.1016/S0140-6736(08)61669-4 18994650

[24] Mitchell RJ, Richardson EA, Shortt NK, Pearce JR. Neighborhood environments and socioeconomic inequalities in mental well-being. Am J Prev Med. 2015;49(1):80-84. doi:10.1016/j.amepre.2015.01.017 25911270

[25] Frieden TR A framework for public health action: the health impact pyramid. Am J Public Health. 2010;100(4):590-595. doi:10.2105/AJPH.2009.185652 20167880 PMC2836340

[26] Gong Y, Palmer S, Gallacher J, Marsden T, Fone D A systematic review of the relationship between objective measurements of the urban environment and psychological distress. Environ Int. 2016;96:48-57. doi:10.1016/j.envint.2016.08.019 27599349

[27] Branas CC, Cheney RA, MacDonald JM, Tam VW, Jackson TD, Ten Have TR A difference-in-differences analysis of health, safety, and greening vacant urban space. Am J Epidemiol. 2011;174(11):1296-1306. doi:10.1093/aje/kwr273 22079788 PMC3224254

[28] Garvin EC, Cannuscio CC, Branas CC Greening vacant lots to reduce violent crime: a randomised controlled trial. Inj Prev. 2013;19(3):198-203. doi:10.1136/injuryprev-2012-040439 22871378 PMC3988203

[29] Branas CC, Kondo MC, Murphy SM, South EC, Polsky D, MacDonald JM Urban blight remediation as a cost-beneficial solution to firearm violence. Am J Public Health. 2016;106(12):2158-2164. doi:10.2105/AJPH.2016.303434 27736217 PMC5104992

[30] Branas CC, South E, Kondo MC, . Citywide cluster randomized trial to restore blighted vacant land and its effects on violence, crime, and fear. Proc Natl Acad Sci U S A. 2018;115(12):2946-2951. doi:10.1073/pnas.1718503115 29483246 PMC5866574

[31] Campbell MK, Piaggio G, Elbourne DR, Altman DG; CONSORT Group CONSORT 2010 statement: extension to cluster randomised trials. BMJ. 2012;345:e5661. doi:10.1136/bmj.e5661 22951546

[32] Boruch R, May H, Turner H, Estimating the effects of interventions that are deployed in many places: place-randomized trials. Am Behav Sci. 2004;47(5):608-633. doi:10.1177/0002764203259291

[33] Schulz KF, Grimes DA Generation of allocation sequences in randomised trials: chance, not choice. Lancet. 2002;359(9305):515-519. doi:10.1016/S0140-6736(02)07683-3 11853818

[34] American Association for Public Opinion Research AAPOR response rate calculator. https://www.aapor.org/Education-Resources/For-Researchers/Poll-Survey-FAQ/Response-Rates-An-Overview.aspx. Accessed November 27, 2017.

[35] Groves RM Nonresponse rates and nonresponse bias in household surveys. Public Opin Q. 2006;70(5):646-675. doi:10.1093/poq/nfl033

[36] Galea S, Tracy M. Participation rates in epidemiologic studies. Ann Epidemiol. 2007;17(9):643-653. doi:10.1016/j.annepidem.2007.03.013 17553702

[37] Keeter S, Kennedy C, Dimock M, Best J, Craighill P Gauging the impact of growing nonresponse on estimates from a national RDD telephone survey. Public Opin Q. 2006;70(5):759-779. doi:10.1093/poq/nfl035

[38] Kessler RC, Andrews G, Colp LJ, Short screening scales to monitor population prevalences and trends in non-specific psychological distress. Psychol Med. 2002;32(6):959-976. doi:10.1017/S0033291702006074 12214795

[39] Kessler RC, Barker PR, Colpe LJ, . Screening for serious mental illness in the general population. Arch Gen Psychiatry. 2003;60(2):184-189. doi:10.1001/archpsyc.60.2.184 12578436

[40] Cohen J Statistical Power for the Behavioral Sciences. 2nd ed Hillsdale, NJ: Lawrence Erlbaum Associates; 1988.

[41] Meyer BD Natural and quasi-experiments in economics. J Bus Econ Stat. 1995;13(2):151-161. doi:10.1080/07350015.1995.10524589

[42] US Department of Health and Human Services, Assistant Secretary for Planning and Evaluation Poverty guidelines. https://aspe.hhs.gov/2013-poverty-guidelines#thresholds. Accessed April 20, 2018.

[43] Branas CC, Macdonald JM A simple strategy to transform health, all over the place. J Public Health Manag Pract. 2014;20(2):157-159. doi:10.1097/PHH.0000000000000051 24458312 PMC3992082

[44] Kondo MC, South EC, Branas CC. Nature-based strategies for improving urban health and safety. J Urban Health. 2015;92(5):800-814. doi:10.1007/s11524-015-9983-y 26275455 PMC4608934

[45] Woolf SH, Braveman P Where health disparities begin: the role of social and economic determinants—and why current policies may make matters worse. Health Aff (Millwood). 2011;30(10):1852-1859. doi:10.1377/hlthaff.2011.0685 21976326

[46] Kuo FE, Sullivan WC Aggression and violence in the inner city: effects of environment via mental fatigue. Sage Journals. 2001;33(4):543-571. doi:10.1177/00139160121973124

[47] Kaplan S The restorative benefits of nature: toward an integrative framework. J Environ Psychol. 1995;15(3):169-182. doi:10.1016/0272-4944(95)90001-2

[48] Hartig T, Evans GW, Jamner LD, Davis DS, Gärling T Tracking restoration in natural and urban field settings. J Environ Psychol. 2003;23(2):109-123. doi:10.1016/S0272-4944(02)00109-3

[49] Ward Thompson C, Roe J, Aspinall P, Mitchell R, Clow A, Miller D More green space is linked to less stress in deprived communities: evidence from salivary cortisol patterns. Landsc Urban Plan. 2012;105(3):221-229. doi:10.1016/j.landurbplan.2011.12.015

[50] Cohen DA, Inagami S, Finch B The built environment and collective efficacy. Health Place. 2008;14(2):198-208. doi:10.1016/j.healthplace.2007.06.001 17644395 PMC2684872

[51] Maas J, van Dillen SME, Verheij RA, Groenewegen PP Social contacts as a possible mechanism behind the relation between green space and health. Health Place. 2009;15(2):586-595. doi:10.1016/j.healthplace.2008.09.006 19022699

[52] Kweon B-S, Sullivan WC, Wiley AR Green common spaces and the social integration of inner-city older adults. Sage Journals. 1998;30(6):832-858. doi:10.1177/001391659803000605

[53] Kuo F, Sullivan W, Coley R, Brunson L Fertile ground for community: inner-city neighborhood common spaces. Am J Community Psychol. 1998;26(6):823-851. doi:10.1023/A:1022294028903

[54] Sullivan WC The fruit of urban nature: vital neighborhood spaces. Sage Journals. 2004;36(5):678-700. doi:10.1177/0193841X04264945

[55] Fone D, White J, Farewell D, . Effect of neighbourhood deprivation and social cohesion on mental health inequality: a multilevel population-based longitudinal study. Psychol Med. 2014;44(11):2449-2460. doi:10.1017/S0033291713003255 24451050

[56] Kruger DJ, Reischl TM, Gee GC. Neighborhood social conditions mediate the association between physical deterioration and mental health. Am J Community Psychol. 2007;40(3-4):261-271. doi:10.1007/s10464-007-9139-7 17924185

[57] Fone D, Dunstan F, Lloyd K, Williams G, Watkins J, Palmer S Does social cohesion modify the association between area income deprivation and mental health? a multilevel analysis. Int J Epidemiol. 2007;36(2):338-345. doi:10.1093/ije/dym004 17329315

[58] de Vries S, van Dillen SME, Groenewegen PP, Spreeuwenberg P Streetscape greenery and health: stress, social cohesion and physical activity as mediators. Soc Sci Med. 2013;94:26-33. doi:10.1016/j.socscimed.2013.06.030 23931942

[59] Triguero-Mas M, Dadvand P, Cirach M, . Natural outdoor environments and mental and physical health: relationships and mechanisms. Environ Int. 2015;77:35-41. doi:10.1016/j.envint.2015.01.012 25638643

[60] Sugiyama T, Leslie E, Giles-Corti B, Owen N Associations of neighbourhood greenness with physical and mental health: do walking, social coherence and local social interaction explain the relationships? J Epidemiol Community Health. 2008;62(5):e9. doi:10.1136/jech.2007.064287 18431834

[61] Stafford M, Chandola T, Marmot M Association between fear of crime and mental health and physical functioning. Am J Public Health. 2007;97(11):2076-2081. doi:10.2105/AJPH.2006.097154 17901443 PMC2040373

[62] Heckert M, Kondo M. Can “cleaned and greened” lots take on the role of public greenspace? J Plan Educ Res. 2017;38(2):211-221. doi:10.1177/0739456X16688766.

