# Error in Figure

**DOI:** 10.1001/jamanetworkopen.2018.2583

**Published:** 2018-08-17

In the Original Investigation titled “Effect of Greening Vacant Land on Mental Health of Community-Dwelling Adults: A Cluster Randomized Trial,”^1^ published July 20, 2018, there was an error in Figure 2B. The panel label should have read “Greening intervention.” This article was corrected online.
