# Supplementary material for: Effect of Greening Vacant Land on Mental Health of Community-Dwelling Adults: A Cluster Randomized Trial
Source: JAMA Netw Open. 2018 Jul 20;1(3):e180298. doi: 10.1001/jamanetworkopen.2018.0298 (PMC6324526; doi:10.1001/jamanetworkopen.2018.0298)
Supplement: Supplement. — Trial Protocol [file jamanetwopen-1-e180298-s001.pdf]

Title: A randomized trial of urban vacant lot stabilization

1. Research design and methods

Study site and design

We are proposing a community intervention trial of urban vacant lot stabilization. The treatment (vacant lot stabilization) would be randomly assigned to vacant lots (e.g. akin to a water fluoridation trial) and would be a primary prevention strategy intended to prevent high risk behaviors.[1] The proposed trial will use a stratified randomization of vacant lots into full treatment, trash clean-up, and no treatment arms matched within 4 sections of Philadelphia: north, south, west/southwest, and northwest. These sections of Philadelphia have clearly delineated roadway and water boundaries. Northeast Philadelphia is excluded because of the very limited number of vacant lots.

Vacant lots will serve as the index locations of data collection for the trial and its outcomes. Outcomes will be surveyed around each vacant lot. Study groups (treatment and controls) will be concurrently exposed to the intervention, or not, within the same 3 month period, with the study following a parallel group trial design where each vacant lot receives only one treatment. Treatment or control status will be assigned to randomly selected lots within the same four sections of Philadelphia, i.e. matched by section, to promote comparability among study groups. The treatment period must coincide with one of two planting seasons, either in the spring or in the fall to properly implement the treatment. All vacant lots will be randomly assigned to either be stabilized (full treatment), have trashed cleaned up (trash control), or have nothing done (no treatment).

The four sections of Philadelphia that will be involved encompass the vast majority of the city and its population. Across these four sections of Philadelphia, a total of 525 vacant lots will be studied. These 525 lots will each be roughly 1000 square feet in area on average and will be clustered in groups of five lots (called sites) since it is standard practice for the PHS to treat lots in clusters of five in order to maximize impact. Thus, across all four sections of Philadelphia there will be a total of 525 lots in 105 sites; each arm of the study (full treatment, trash control, and no treatment) will have 175 lots in 35 sites.

Proposed treatment to be tested (full treatment arm)

We propose to perform full vacant lot stabilizations on 175 randomly selected vacant lots in 4 of the 5 sections of Philadelphia. Each of these vacant lots will be approximately 1000 square feet in area. A significant portion of the City of Philadelphia will thus be touched by the actual treatment in the proposed trial. This full treatment will be performed by the PHS and its contractors. (a letter from the PHS is included in the Appendix). The PHS and its landscape contractors are accustomed to performing vacant lot stabilizations on geographical units with areas of 80,000 to 200,000 square feet per spring and fall planting seasons each year.

Vacant lots will be stabilized in groups of five as opposed to single lots. Each group of five lots will be either immediately contiguous or within 660 feet (a standard distance denoting close proximity in Philadelphia City ordinances) of its nearest neighbor vacant lot. Each group of five lots will be labeled a “site” and will be roughly 5000 square feet in total area. The PHS typically does their regular lot stabilizations in these five-lot groupings because it is thought to be much more effective than the stabilization of isolated, singular lots. All square footage in each site will be stabilized.

The proposed trial will only treat vacant lots that the PHS is authorized to stabilize as per the Philadelphia Department of Licenses and Inspections. This is exactly the same authorization procedure that PHS uses in its day-to-day vacant lot stabilization program outside the proposed trial. These authorized lots will constitute
the vast majority of lots in the four sections of Philadelphia. Authorized lots will: (1) have existing violations
signaling “blight”, including illegal dumping, abandoned cars, and/or unmanaged vegetation growth (greater

than 14 inches high); and (2) have been abandoned, as confirmed by a call to the owner of record who is given 10 days to reply and offers no reply in said time; or (3) have been authorized for stabilization by the lots owners themselves within the 10 day period (as they want the free greening treatment). The vast majority of vacant lots in the universe of vacant lots in the four sections of Philadelphia we will study will fall into one or more of these categories and be available for enrollment in the trial. Lots that have already been remediated by the PHS or other local or municipal agencies will not be eligible for enrollment. We will pre-screen to determine this universe of candidate vacant lots for enrollment.

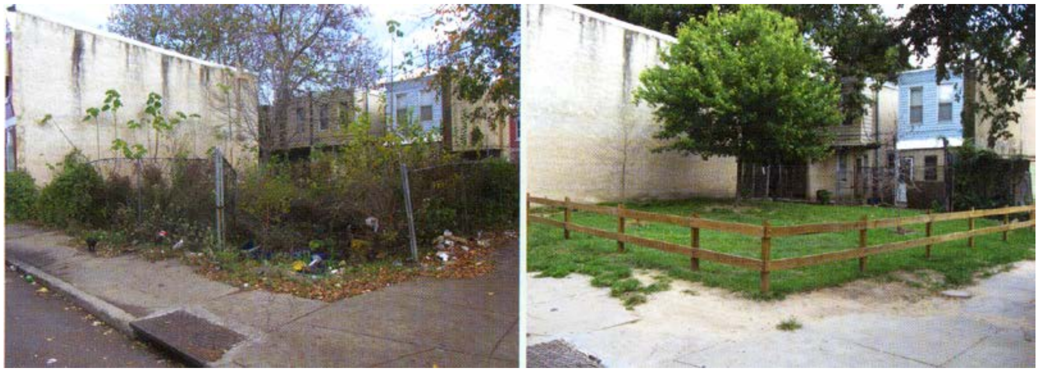

Figure. Before (left) and after (right) vacant lot stabilization treatment by the Pennsylvania Horticultural Society in Philadelphia.

The proposed vacant lot stabilization treatment will involve “cleaning and greening” abandoned vacant lots by removing debris, grading the land, planting grass and trees to create a park-like setting, and installing low wooden fences around each lots’ perimeter to show that the lot is cared for and to deter illegal dumping. The PHS has decades of experience with this vacant lot treatment in Philadelphia. Although this stabilization process is simple, its results are visually dramatic (see Figure) and have been shown to boost housing values.[2] The treatment also includes regular maintenance, grass cutting, tree pruning, fence repair, and trash clean-up in the post-treatment period.

Proposed trash clean-up control to be tested (trash control arm)

As a control arm of the proposed trial, we will also randomly select a separate group of 175 vacant lots across the same four sections of Philadelphia to receive trash clean-up only. These control lots will not receive the actual full vacant lot stabilization treatment. This control group of vacant lots will receive their trash clean-up in the same planting season as the full treatment group of vacant lots and then also receive regularly scheduled maintenance clean-ups (to match the full treatment group) over the same post-treatment period.

Uncontrolled trials fail to provide unbiased and reliable statistical inference regarding what would have happened to subjects if they had not received the test treatment.[3] Our proposed use of a randomly selected trash clean-up control group of vacant lots will be akin to a “placebo” group in a clinical trial, that is it will be intended to eliminate observer bias, mimic the psychological benefit of offering active treatment[4], and allow isolated study of the nonpsychological benefits of treatment.[5] The proposed trash clean-up control group will allow us to disentangle the psychological effects of vacant lot stabilization and the act of vacant lot stabilization as opposed to the actual active ingredient of vacant lot stabilization, the greening itself (defined as grading the land, planting grass and trees to create a park-like setting, and installing low wooden post-and-rail fences).

Each “trash clean-up only” control vacant lots will be approximately 1000 square feet in area, the same as the full treatment group of vacant lots. And, just as with the full treatment group of vacant lots, a significant portion of Philadelphia will be touched by this proposed trash clean-up. This “trash clean-up only” control will also be performed by the PHS and its contractors. Control vacant lots will have trash removed from them in groups of five, the same as the full treatment group of vacant lots. Again, each group of five lots will be either immediately contiguous or within 660 feet of its nearest neighbor vacant lot. Each group of five lots will be labeled a “site”.

All square footage in each site will be cleaned of trash. The proposed trial will only remove trash from vacant lots for which the PHS is authorized in its day-to-day program outside the trial.

Proposed control treatment to be tested (no treatment arm)

As a second control arm of the proposed trial, we will also randomly select a separate group of 175 vacant lots in four of the five sections of Philadelphia to receive nothing, i.e. no vacant lot stabilization and no trash clean-up. This control group of vacant lots will be monitored during the same planting season and for the same post-period exactly as with the full treatment group of vacant lots and the trash clean-up group of lots.

Although "no treatment" controls fail to simulate the psychological effect of treatment, they are important when used in conjunction with controls that do simulate such psychological effects of treatment.[6] Our proposed use of a randomly selected control group of vacant lots to receive nothing is therefore intended to eliminate observer bias and other selection effects, when compared with the other two arms of the trial.[7]

Each "no treatment" control vacant lot will be approximately 1000 square feet in area, the same as the full treatment group of vacant lots. These control vacant lots will also be considered in groups of five as opposed to single lots, the same as the full treatment group of vacant lots. Again, each group of five lots will be contiguous or within 660 feet of its nearest neighbor vacant lot. Each group of five lots will be labeled a "site". These "no treatment" vacant lots will also be lots that the PHS would have been authorized to stabilize. We will rely on exactly the same authorization procedure that the PHS uses in its day-to-day program. We also recognize that treatment spillover or diffusion of treatment onto control group sites may be a threat to internal validity[8] and we will accordingly explore solutions to counter this based on prior community trials in Philadelphia[9], such as requiring that treatment and control sites are no closer than some minimum distance. However, this issue would likely tend to equalize the outcomes between treatment and control groups, producing, if anything, conservative estimates of a treatment effect.[10]

Randomization procedures

The randomization of lots to 3 different arms of the trial will be done to balance known and unknown factors between treatment and control groups.[11] A randomization list will be generated and will include lot ID numbers, random assignments, and parallel groupings or strata. Once the trial starts, vacant lots meeting the inclusion criteria will sequentially get their random assignments based on randomization codes on a predetermined list. Randomization codes will be securely filed with the study's biostatistician to maintain blinding.

As part of a stratified randomization strategy, the randomization of lots to the treatment and control groups will be performed independently within strata by geographic section. This will keep the variability of lots within strata as small as possible and the between-strata variability as large as possible so that the inference for the treatment effect possesses the optimal precision. This will also prevent imbalance with respect to important covariates related to geographic location (e.g., neighborhood socioeconomic status, race, ethnicity, alcohol sales, illicit drug markets, etc.). For example, in multicenter trials, stratified randomization with respect to geographical location is necessary because differences in study centers usually account for the major source of variation for many primary endpoints. This same geographic stratification will be needed for the community trial proposed here and will take the form of geographic sections of Philadelphia as strata. In this way, geographic sections of Philadelphia will encompass and represent covariates that are absolutely necessary for the integrity of the study and, as such, will be part of the proposed stratified randomization. We will also additionally explore the stratification of lots on other important covariates although, as per established standards, the trial we are proposing will be sufficiently large enough to promote balanced allocation within strata and, as such, the logistic challenge of implementing these stratifications may not be necessary.[12,13,14,15]

In implementing the proposed trial's randomization, standard procedures for generation, implementation, and administration of randomization will be used. Lots will be randomized on a 1:1 basis to one of the three study groups. Block randomization with block sizes ranging randomly between 4 and 8 consecutive lots will be employed to ensure that equal numbers of lots

are assigned to each of the two groups and are balanced with respect to observed and unmeasured baseline, temporal, and spatial factors, including methods of measurement of observations.[16] This randomization method employed for the study will be detailed in a User's Reference Manual that will be developed and will contain detailed instructions for the use of the system, references to the pseudorandom number generator, methods of randomization, programs for listings of the pseudo-random number generator and for the production of a listing of the randomization codes. Once randomization codes undergo acceptable quality assurance checks with the trial's biostatistician, the study treatments and controls will be implemented. Information of the randomization codes will then be locked in a database until interim or final analyses need to be performed. In addition, several dry runs with simulated cases will be done before the actual implementation of the system takes place.

Blinding procedures

Blinding, or masking, will be employed to avoid the risk of personal bias in comparing treatments caused by subjective judgment in reporting, evaluation, data processing, and statistical analysis. For the proposed trial, we will employ double blinding, that is our investigative team (except for the biostatistician and the PI) and field surveyors, and our participants completing surveys and those reporting information to us from the field (such as the Philadelphia Police Department) will be blinded to the assignment of treatment or control to vacant lots. This will help avoid bias created by participant or field interviewer awareness of nearby vacant lot stabilization.

Although this double blinding may be eroded given that both participants and field investigators may see a lot that has been stabilized near them, we will not confirm that this is the case if they ask (because the field investigators will be blinded, if they are asked by a participant about a specific lot near their house they will truly not be able to confirm whether this lot was greened as part of the trial). We will also determine whether the blinding has been seriously violated by asking both participants and investigators to guess the treatment assignment at the conclusion of the trial prior to unblinding. With this information the degree of unblinding and its impact on introducing bias in the evaluation of treatment effect can be directly assessed.

2. Outcomes data to be collected

Observed indicators of crime

Crime data will be collected from the Philadelphia Police Department every month in the year preceding the treatment period, for the treatment period itself, and for 12 months following the treatment period. We will thus have 12 pre-treatment and 12 post-treatment time points. Police Department data will include dates and address locations of violent crimes, narcotics possession, sales, and trafficking arrests, public drunkenness, and other crimes. These arrest data have been validated as accurate proxies of actual drug trafficking, sales, and use in urban environments.[17] Address locations will be geographically coded ("geocoded") to points in space using standard geographic information systems (GIS) software. The Philadelphia Police Department has developed a GIS infrastructure that is one of the largest distributed, integrated municipal GISs in the US.

We will also use inverse-distance weighted (IDW) measures to calculate relatively straightforward, spatially interpolated estimates of the levels of crime at the point-in-space representing each vacant lot.[18] We will also incorporate bandwidths (maximum distances beyond which crimes will no longer be considered) for our IDW measures as is standard practice. IDW measures have a long history of use by geographers and will offer our analysis several important advantages over simply assigning subjects to solitary geographic polygons, such as census tracts or block groups. Analyses in which subjects are nested within solitary administrative geographic units (i.e., a single census tract or block group) can generate challenges, including the mis-estimation of effects. Oftentimes, the boundaries of these administrative geographic units have been determined for purposes other than the specific relationships under study and as such may be awkwardly shaped, poorly correspond to lived space, have edge-effects (i.e., a subject assigned to a tract but located on its border may be more influenced by their neighboring tract), or impose a neighborhood scale that is inappropriate for the subjects being studied. For these reasons, we propose to use IDW measures, which are continuous and essentially boundary-free, essentially

assign each lot their own unique neighborhood, avoid aggregation effects, and directly account for spillover effects and the variability of neighboring areas. Our current experience in Philadelphia with such measures is that they can be quite reliably collected.[19,20]

Recurring field observations and trash censuses will also be conducted for all study lots, with specific counts of drug and alcohol related objects (cans, bottles, syringes, vials, etc.) found on-site. These field observations will be conducted monthly, according to a systematic protocol. They will be coordinated with the PHS's lot cleaning schedule, to occur immediately before any clean-up efforts, and to allow a calibration between what is observed and the actual trash collected. Pictometric measures of trash and drug and alcohol related objects will be deployed and analyzed using the protocols developed in our preliminary studies. Our current experience in Philadelphia with such measures is that they can be quite reliably collected.[21,22]

Self-reported indicators of health and safety
Participant recruitment and incentives

We will recruit 2 randomly selected Philadelphia residents over 18 years old to complete a survey of health and safety for the 105, 5000 square foot vacant lot sites across all three arms of the proposed trial. This will be a total of 210 residents to be surveyed for the trial as a whole. These residents will live near the index vacant lot to which they will be assigned. Residents over 18 years old have been chosen because they are not minors but to also include 18-20 year olds who will not be legal to drink alcohol and therefore, most likely to engage in and be familiar with surreptitious alcohol consumption practices, likely outdoors. These residents will be asked to complete the same survey twice in the year before and twice in the year after the 3 month treatment period during which the vacant lot near them has either been fully treated, had trashed removed, or had nothing done.

We will pay these residents a total of $50 each as fair incentive for their time. This incentive payment will be distributed as $10 for the first interview, $10 for the second interview, $10, for the third interview, and $20 if they complete the final interview. This increasing incentive structure is designed to maximize participant retention and minimize attrition. We anticipate that the survey will take 15-20 minutes each time and it will be constructed such that subsequent interviews (after the first interview which will be face-to-face) can be completed over the telephone. Because the method of self-reported data collection will switch, final data analyses will be structured to statistically account for this with an variable indicating whether each survey was completed face-to-face or over the telephone. A loss-to-follow up / dropout rate of about 25% is assumed, based on our prior experience conducting such face-to-face and telephone surveys in the field in Philadelphia.[23,24,25] This translates into a total of 280 participants (210 / 75%; or 2.67 respondents per vacant lot site) that will be needed to fully obtain 2 completed respondents per vacant lot site. We will thus recruit and consent 3 participants per vacant lot site to conservatively insure that at least two of these participants will complete the four repeat interviews.

A longitude-latitude (X-Y) coordinate point location will be generated within the boundaries of each vacant lot site. This will be the centroid point location of the vacant lots calculated from the polygon that represents the boundaries of the site itself. The location of the closest building or structure address will then be determined for this point location. A survey team will physically go to the building or structure closest to each randomly selected point location. This team will carry hand-held global positioning systems (GPS) units. At each address, surveyors will walk in a random direction (as determined by GPS) until they have completed a total of two interviews in two separate households. The first household will be surveyed because it is the closest to the address corresponding to the randomly selected point location. Subsequent households will then be similarly surveyed by proximity. Thus, from a start household, the survey team will proceed to each adjacent household until a total of two interviews in two separate households are completed.

Within each randomly selected household, participant consent and a "household census" will initially be conducted. This will determine eligibility of occupants (and screen out ineligible occupants, such as minors less than 18 years old) and their willingness to participate in the study. In the event that a household is identified with multiple eligible respondents who consent to participate, we will select the respondent whose birthday is closest to the date of the interview. If

multiple families are living in the same building, they will be regarded as one household unless they each have separate entrances onto the street.

Households that refuse to participate, cannot participate, or do not qualify to participate in the survey will be marked as such and the surveyors will move on to the next closest household. Surveyors will revisit empty (but not abandoned) households later in the day or the next morning, and ask for the help of neighbors to trace absentees. Households with no one home after five attempts will be skipped and not revisited.

Although every attempt will be made to conduct an in-person interview with each randomly sampled subject, proxy respondents will be used when the sampled person is incapable of completing the interview (for instance, due to health reasons or unavailability for the entire study period). In some instances, the sample person may require an assistant for either part or the entire interview. We will control for any confounding effects of these proxies by assessing the effect of proxy status as a confounder in each of our models.

Survey administration

The survey will be administered in English (or Latin Spanish), as appropriate. The proposed study's field survey team will consist of a team leader/project coordinator and preferably one male and one female interviewer. Interviewers will be trained ahead of time in the consistent and appropriate administration of face-to-face and telephone surveys in general and our survey in particular. Before implementing the full survey on actual respondents, we will test it for readability, timing, logic, flow, and burden on a series of pilot subjects.

If the interview team feels threatened or in danger due to their surroundings or the respondent they are interviewing, the interview will be stopped and the team will leave the area. As with other field interviews that we have conducted in Philadelphia, interviewer safety will be of the utmost importance. Interview teams will carry only enough incentive money to remunerate at most two respondents, to avoid being targets for crime.

All interviews will be respectfully administered and interviewers will explain questions and respond to any concerns the respondents might have over the course of the interview. This will allow us to establish excellent rapport with respondents and assure them that we are researchers with a private, local university and in no way affiliated with law enforcement or government agencies. Consent to conduct the survey will be obtained before any actual survey questions are asked. Potential respondents that refuse will not be asked further questions. No analyses, reports, or peer-reviewed articles will identify any participants. Interview may also be conducted somewhere nearby but not in the respondent's household, such as a church, park, café, etc.

We will record information about the participant on the first interview that will allow us to recontact that same individual for three subsequent interviews. With the participant's consent, this information may include address and/or telephone number so that we can recontact them in subsequent interviews, possibly over the telephone. Participants will also be informed that any other identifying information that is recorded – such as names, birthdates, etc. – for study participants, will be destroyed at the conclusion of the study.

Survey domains and measurement

The survey itself will primarily ask respondents about their observations of public behaviors such as drug and alcohol use in their immediate vicinity, as well as their own drug and alcohol use behaviors, both indoors and outdoors. The survey questionnaire itself will be separated into the following sections – Introduction, Background, Neighborhood Observations of Public Substance Use and Sales (Alcohol and Drugs), Personal Substance Use and Sales (Alcohol and Drugs), Crime, Mental Health, Trauma History, Stress and Fear, Closing Remarks, and Interviewer Impressions. Survey questionnaires will be standardized with mostly closed-ended, yes/no, screener questions (that can be answered quickly and with minimal burden to respondents) and some open-ended questions and opportunities for respondents to provide narrative responses. For respondents who have a difficult time recalling events, we will use a calendar of locally important events to anchor their memory and facilitate recall and we will also give them clear dates and/or events to bound their recall and avoid reporting of events outside of the 6 month reference period. This avoids errors due to telescoping which can lead to over-estimations. Questions that have been reliably implemented in other settings and in Philadelphia

will be drawn from standardized questions taken from our own prior survey work of substance abuse and the urban environment [26,27,28,29] and sources like well-studied substance abuse and quantity-frequency consumption questions[30,31,32,33], well studied neighborhood environment questions from Philadelphia[34,35] and other cities like the Project on Human Development in Chicago Neighborhoods Survey[36], the Trauma History Questionnaire[37], the Primary Care Stress Questionnaire[38], the National Health Interview Survey[39], and portions of other standardized survey instruments that will be considered for inclusion during the planning phase of the trial. As detailed above, these survey instruments have been shown to be reliable in prior work, both our own and that of others.

3. Analysis strategy

Statistical Analyses

Standard descriptive statistics will be used to analyze baseline and compare before-after factors in all three arms of the proposed trial. Summary statistics such as means, medians, standard deviations, skewness and kurtosis will be produced for measured variables. Frequencies will be tabulated for categorical and ordinal variables. Graphical methods, including mapping, will be used extensively to examine distributions in time and space and identify potentially influential points. Scatter plots and grouped box plots will be produced to examine associations and assumptions of linearity, symmetry, and heteroscedasticity.

Our primary analyses will be performed under the intent-to-treat (ITT) principle (i.e., vacant lots will be analyzed according to the treatment group to which they were randomly allocated), comparing the pre-post changes of the randomized treatment and control groups. For the primary ITT hypotheses, tests of the randomized group differences in pre- versus post-intervention temporal change for continuous, count, or binary (or ordinal) outcomes will be made using random effects linear, log-linear or logistic models, respectively. Such models will include random effects for temporal and spatial correlations and fixed effects for: 1) the greening/control factor; 2) time effects characterizing changes across time for each of the pre- and post-intervention periods; and 3) the interaction between the greening/control factor and the pre- and post-intervention temporal effects. Tests and estimation of this interaction will represent the ITT randomized group contrast in pre- versus post-intervention change in outcome. Such models will be employed for each lot outcome and each participant mental and physical performance outcome, dichotomized or continuous as appropriate. As a secondary analysis, any baseline or temporal factors that are statistically significantly different between groups will be included in the primary ITT models to adjust for any “residual” confounding not prevented by the randomization. Given the large number of randomized lots, it is unlikely that we will have baseline factors or temporal factors that differ between baseline groups. Group differences for these factors will be assessed using random effects linear, log-linear or logistic models for continuous, count, or binary (or ordinal) outcomes, respectively. We will include random effects to control for temporal and spatial correlations.

Additional analyses will include as-treated analyses of the association between each pre- and post-treatment change variable. We define ”as-treated” to be any lot randomized to greening that actually receives the greening intervention, but not “as-treated” to be any lot randomized to greening that does not actually receive greening for some reason. For this analysis, the primary ITT analysis random effects models will include as the “treatment” factor the “as-treated” status variable rather than the randomized assignment as under the ITT analysis. We will also assess whether the ITT and as-treated effects vary across the different sections of the city by including section-treatment interaction effects and section main effects In the above models, and testing the interaction parameters. All models will be fit using Proc Glimmix in SAS V9.2 based on the modeling procedures in Brown et al. as a special case of multiple-membership classification models (accounting for spatial clusters of nearby lots). Missing data weights based on inverse probability weighting will be used as a sensitivity analysis if the missing data are significantly related to baseline factors.[40]

Sample size

We present sample sizes for different levels of power and different numbers of time points for the lot-specific primary aggregate assault count outcome based on the group-pre vs. post

interaction test for any pairwise comparison among the three randomized groups of lots. We make the following assumptions[41]:

1) two-sided alpha = 0.0167 to control for pairwise comparisons
2) standard deviation of the drug case counts/mile = 660 and of drunkenness cases = 14.3/ mile;
3) clinically significant effect size is 0.20 (= interaction parameter/std) leading to group-pre vs. post interaction = 132 for drug cases/mile and 2.86 for drunkenness cases/mile;
4) no missing data since complete drug and alcohol data are available;
5) within-lot time correlation ($rho(t)_y$) for drug or alcohol data = 0.40; and
6) between-lot spatial correlation ($rho(s)_y$) for assault counts = 0.10; and
7) within-lot correlation (rho_x) for the -1,1 dummy variables of group & pre-post indicator variables = -0.01.

Using the design effect formula, $(1+(T-1)*rho_x*rho(t)_y)$, where T is the number of time points and $(1+ (K-1)*rho(s)_y)$, where K is the average number of lots within a cluster of near lots [42] effective sample sizes accounting for the above within-lot correlations (temporal relationships) were calculated from simple random sample sizes in PASS. According to Kraemer et al.[43] (2006), we base the Cohen's effect size on a clinically meaningful effect size of 0.20, where the effect size is the interaction parameter divided by the standard deviation of the outcome. Table 3 displays sample size results for different frequencies of time period measurements and different levels of statistical power. We did not account for stratifying on the geographic sections of Philadelphia, as this would have only improved power; thus our sample size estimates are conservative In achieving the proposed trial's primary aim of studying the occurrence of public occurrences of drug and alcohol use we will study 175 lots (in 35 separate sites of 5 lots each) in each arm of the trial (full treatment, trash control, and no treatment) to maintain 90% statistical power.

	**Statistical power**				
**Time periods**	**70%**	**75%**	**80%**	**85%**	**90%**
12 pre- and 12 post periods (every 4 weeks)	110 lots (in 22 sites)	125 lots (in 25 sites)	140 lots (in 28 sites)	155 lots (in 31 sites)	175 lots (in 35 sites)
17 pre- and 17 post periods (every 3 weeks)	75 lots (in 15 sites)	85 lots (in 17 sites)	95 lots (in 19 sites)	105 lots (in 21 sites)	120 lots (in 24 sites)
26 pre- and 26 post periods (every 2 weeks)	45 lots (in 9 sites)	50 lots (in 10 sites)	55 lots (in 11 sites)	65 lots (in 13 sites)	70 lots (in 14 sites)
52 pre- and 52 post periods (every week)	15 lots (in 3 sites)	20 lots (in 4 sites)	20 lots (in 4 sites)	25 lots (in 5 sites)	25 lots (in 5 sites)

Table. Sample size calculations for different levels of power and frequencies of time period measurement

We calculated the minimally detectable effect size given 80% power for the participant-level outcomes and 4 time points based on the group-pre vs. post interaction test for any pairwise comparison among the three randomized groups of lots. By minimally detectable effect size, we mean the smallest Cohen's effect size (group-pre vs. post interaction/standard deviation of outcome) that we can call significant with 80% power under the following assumptions: 1) within- participant correlation (rho_y) for participant-level outcomes = 0.70; 2) within-lot correlation (rho_y) for participant-level outcomes = 0.20; 3) group-pre vs. post interaction = 11 assaults per mile; 4) within-lot correlation (rho_x) for the -1,1 dummy variables for group & pre-post indicator variables = -0.33. Given these assumptions, we used the program RMASS[44] to compute the minimally detectable effect size of 0.50 under a nested random effects model to account for the within-lot and within-participant correlations. This is a medium effect size based on Cohen.[45] Based on this we would maintain 80% power if we randomly surveyed three people per vacant lot site, twice before and twice after the treatment period (4 time points total). This calculation accounts for a 25% loss-to-follow up / dropout rate.

Literature Cited

1 Rothman KJ, Greenland S. Modern Epidemiology. 2nd Edition. 1998 Lippincott-Raven Publishers.

2 Wachter, S. The Determinants of Neighborhood Transformations in Philadelphia - Identification and Analysis: The New Kensington Pilot Study. Wharton School, University of Pennsylvania Working Paper, 2004.

3 Chow S, Liu J. Design and analysis of clinical trials. 2004 John Wiley and Sons, 2nd edition.

4 Rothman KJ, Greenland S. Modern Epidemiology. 2nd Edition. 1998 Lippincott-Raven Publishers.

5 Rothman KJ, Greenland S. Modern Epidemiology. 2nd Edition. 1998 Lippincott-Raven Publishers.

6 Chow S, Liu J. Design and analysis of clinical trials. 2004 John Wiley and Sons, 2nd edition.

7 Rothman KJ, Greenland S. Modern Epidemiology. 2nd Edition. 1998 Lippincott-Raven Publishers.

8 Trochim WMK, Donnelly JP. Experimental Design. Chapter 9 In: Research Methods Knowledge Base, 2007 Thomson Publishing.

9 Ratcliffe, JH, Taniguchi, T, Groff, ER & Wood, JD (in press) The Philadelphia Foot Patrol Experiment: A
randomized controlled trial of police patrol effectiveness in violent crime hotspots. Criminology.

10 Trochim WMK, Donnelly JP. Experimental Design. Chapter 9 In: Research Methods Knowledge Base, 2007 Thomson Publishing.

11 Chow S, Liu J. Design and analysis of clinical trials. 2004 John Wiley and Sons, 2nd edition.

12 Hubbard WK. Associate Commissioner for Policy Coordination. DEPARTMENT OF HEALTH AND HUMAN SERVICES. Food and Drug Administration [Docket No. 97D–0174]. International Conference on Harmonisation; Guidance on Statistical Principles for Clinical Trials; Availability. Federal Register; Vol. 63, No. 179, September 16, 1998.

13 Klar N, Donner A. The merits of matching in community intervention trials: a cautionary tale. Statistics in Medicine 1997;16:1753-1764.

14 Chow S, Liu J. Design and analysis of clinical trials. Concepts and methodologies. 2nd ed., 2004 Wiley and Sons, Inc., Hoboken, New Jersey.

15 Meinert C. Clinical Trials. 1986, Oxford University Press.

16 Piantadosi S. Clinical Trials: A Methodological Perspective Wiley, NY, p. 276-277.

17 Warner BD, Coomer BW. Neighborhood drug arrest rates: Are they a meaningful indicator of drug activity? A research note. Journal of Research in Crime and Delinquency. 2003; 40(2):123-138.

18 Branas CC, Elliott MR, Richmond TS, Culhane D, Ten Have TR, Wiebe DJ. Alcohol consumption, alcohol outlets, and the risk of being assaulted with a gun. Alcoholism: Clinical and Experimental Research 2009; 11(4): 906-915. PMC: 2831053.

19 Branas CC, Cheney RA, MacDonald JM, Tam VW, Jackson TD, Ten Have TR. The health and safety effects of greening vacant urban space: a difference-in-differences analysis. American Journal of Epidemiology 2011; (under review).

20 Branas CC, Elliott MR, Richmond TS, Culhane D, Ten Have TR, Wiebe DJ. Alcohol consumption, alcohol outlets, and the risk of being assaulted with a gun. Alcoholism: Clinical and Experimental Research 2009; 11(4): 906-915. PMC: 2831053.

21 Branas CC, Cheney RA, Wiebe DJ, Cannuscio C. Comparing in-field and publicly available internet photographs of urban space. Journal of Epidemiology and Community Health 2011; (in preparation).
22 Cannuscio CC, Weiss EE, Fruchtman H, Schroeder J, Weiner J, Asch DA. Visual epidemiology: photographs as tools for probing street-level etiologies. Soc Sci Med. 2009 Aug;69(4):553-64.
23 Blackstone MM, Wiebe DJ, Mollen CJ, Kalra A, Fein JA. Feasibility of an interactive voice response tool for adolescent assault victims. Feasibility of an interactive voice response tool for adolescent assault victims.
24 Branas CC, Culhane D, Richmond TS, Wiebe DJ. Novel Linkage of Individual and Geographic Data to Study Firearm Violence. Homicide Studies 2008 Aug;12(3):298-320.
25 Wiebe DJ, Carr BG, Datner EM, Elliott MR, Richmond TS. Feasibility of an automated telephone survey to enable prospective monitoring of subjects whose confidentiality is paramount: a four-week cohort study of partner violence recurrence after Emergency Department discharge. Epidemiol Perspect Innov. 2008 Jan 7;5:1.
26 Blackstone MM, Wiebe DJ, Mollen CJ, Kalra A, Fein JA. Feasibility of an interactive voice response tool for adolescent assault victims. Feasibility of an interactive voice response tool for adolescent assault victims.
27 Branas CC, Culhane D, Richmond TS, Wiebe DJ. Novel Linkage of Individual and Geographic Data to Study Firearm Violence. Homicide Studies 2008 Aug;12(3):298-320.
28 Branas CC, Cheney RA, Wiebe DJ, Cannuscio C. Comparing in-field and publicly available internet photographs of urban space. Journal of Epidemiology and Community Health 2011; (in preparation).
29 Wiebe DJ, Carr BG, Datner EM, Elliott MR, Richmond TS. Feasibility of an automated telephone survey to enable prospective monitoring of subjects whose confidentiality is paramount: a four-week cohort study of partner violence recurrence after Emergency Department discharge. Epidemiol Perspect Innov. 2008 Jan 7;5:1.
30 Soderstrom CA, Dischinger PC, Kerns TJ, et al. Screening trauma patients for alcoholism according to NIAAA guidelines with alcohol use disorders identification test questions. Alcoholism: Clinical & Experimental Research 1998;22:1470-1475.
31 Midanik LT. Validity of self-reported alcohol use: a literature review and assessment. British Journal of Addiction 1988; 83:1019-1029.
32 Cherpitel CJS. Breath analysis and self-reports as measures of alcohol-related emergency room admissions. Journal of Studies of Alcohol 1989; 50(2): 155-161.
33 Soderstrom CA, Smith GS, Kufera JA, et al. The accuracy of the CAGE, the Brief Michigan Alcoholism Screening Test, and the Alcohol Use Disorders Identification Test in screening trauma center patients for alcoholism. Journal of Trauma-Injury Infection & Critical Care 1997;43:962-969.
34 Elo IT, Mykyta L, Margolis R, Culhane JF. Perceptions of neighborhood disorder: The role of individual and neighborhood characteristics. Social Science Quarterly, 2009. 90(5):1298-1320.
35 Cannuscio CC, Weiss EE, Fruchtman H, Schroeder J, Weiner J, Asch DA. Visual epidemiology: photographs as tools for probing street-level etiologies. Soc Sci Med. 2009 Aug;69(4):553-64.
36 Earls F, Visher C. Project on Human Development in Chicago Neighborhoods: A Research Update. NIJ Research in Brief. 1997;163603(9):1-11.
37 Heilemann M, Kury F, Lee K. Trauma and posttraumatic stress disorder symptoms among low income women of Mexican descent in the United States. J Nerv Ment Dis. 2005;193(10):665-672.

38 Prins A, Ouimette P, Kimerling R, Cameron R. The primary care PTSD screen (PC–PTSD): development and operating characteristics. Primary Care Psychiatry. 2004;9:9-14.
39 National Center for Health Statistics. National Health Interview Survey 09.1, 37. 3-17-2009. Hyattsville, MD
40 Browne W, Goldstein H, Rasbash J. Multiple-membership multiple classification (MMMC) models. Statistical Modelling 2001; 1:103-124.
41 Diggle PJ, Liang, KY, Zeger SL. Analysis of Longitudinal Data. 1994 Oxford University Press, New York.
42 Neuhaus JM, Segal MR. Design effects for binary regression models fitted to dependent data. Statistics in Medicine 1993;12: 1259-1268.
43 Kraemer HC, Mintz J, Noda N, Tinklenberg J, Yesavage JA. Caution regarding the use of pilot studies to guide power calculations for study proposals. Archives of General Psychiatry. 2006; 63: 484-489.
44 Roy A, Bhaumik D, Aryal S, Gibbons R. Sample size determination for hierarchical longitudinal designs with differential attrition rates. Biometrics, 2007;63:699-707.
45 Cohen J. Statistical Power for the Behavioral Sciences (2nd ed.). Hillsdale, NJ: Erlbaum, p. 13, 1988.
